# Sustaining Breastmilk Expression and Direct Feeding at Breast for Very Low Birth Infants: A Qualitative Exploration of Parental Perspectives

**DOI:** 10.1111/mcn.70193

**Published:** 2026-05-27

**Authors:** Maria Noonan, Vivienne Fitzgibbon, Rachel Keyes, Jan McCarthy, Carmel Bradshaw, Sylvia Murphy Tighe, Sandra Atkinson, Deirdre O. Connell, Helen Byrt, Mary Shanahan, Joanne Desmond, Barbara Lloyd, Sandra Healy, Roy K. Philips

**Affiliations:** ^1^ School of Nursing and Midwifery University of Limerick Limerick Ireland; ^2^ Health Research Institute (HRI) University of Limerick Limerick Ireland; ^3^ University Maternity Hospital Limerick (UMHL) Limerick Ireland; ^4^ Public Health Nursing Service, HSE Mid‐West Limerick Ireland; ^5^ Department of Paediatrics, Division of Neonatology University Maternity Hospital Limerick (UMHL) Limerick Ireland; ^6^ University of Limerick School of Medicine Limerick Ireland

**Keywords:** breast milk, breastfeeding, direct feeding at breast – DFAB, human milk, milk expression, qualitative methods, very low birthweight infants

## Abstract

In Ireland, while most women express milk for their preterm very low birthweight (VLBW) infants, only a few are discharged home exclusively receiving mothers' own milk, and even fewer are engaged in direct feeding at breast (DFAB). This is in the context of Ireland having one of the lowest breastfeeding initiation and sustenance rates in the world. This qualitative descriptive study aimed to explore parents' experiences and perspectives on sustaining breastmilk expression and DFAB for VLBW infants. Semi‐structured interviews were conducted with nine mothers and three partners, and the data were analysed using Braun and Clarkes' (2022) framework for reflexive thematic analysis. The themes developed were: (1) Sustaining breastmilk expression for preterm infants; (2) Navigating the transition to DFAB; and (3) The emotional Journey of feeding a VLBW infant. Supportive conditions for sustained milk expression included access to equipment, skin‐to‐skin contact, and both practical and emotional support from partners and healthcare professionals. Key influences on the transition to DFAB encompassed infant‐related challenges, health system factors and the role of partner involvement. Scheduled support from lactation consultants, midwives and nurses in both hospital and community settings, who have undertaken specialised training on supporting women to breastfeed a VLBW infant is recommended to facilitate the transition to DFAB. Participants identified strategies that could be integrated into a ‘*DFAB care pathway’* to provide a structured and consistent evidence‐based approach to support women, their premature infants and families.

## Summary

- Parents require personalised, realistic information about the importance and process of expressing breast milk for their preterm very low birth weight infant.
- Scheduled expert lactation support in hospital and community settings can help mothers sustain long‐term milk expression.
- Introduce the concept of direct feeding at breast (DFAB) early in the neonatal intensive care journey as a sustainable and achievable feeding option.
- Ensure consistent access to expert lactation support across care settings to promote successful transition to DFAB.
- Provide emotional support to parents throughout the journey of long‐term milk expression and DFAB, recognising that some may benefit from additional psychological support.

## 1 Background

Mother's own milk (MOM) is the optimal nutritional choice for preterm infants (Verduci et al. 2021). Systematic reviews highlight the significant health benefits of human milk (HM) for very preterm (< 32 weeks gestation) infants compared to term infants (Maastrup et al. 2014). Breastfeeding reduces the risk of infection including late‐onset neonatal sepsis, and necrotising enterocolitis (NEC) (Hair et al. 2018; Lucas and Cole 1990; Sullivan et al. 2010). It lowers the rates of retinopathy of prematurity (Bharwani et al. 2016; Zhou et al. 2015) and bronchopulmonary dysplasia (Spiegler et al. 2016). Additionally, HM positively impacts immunologic, nutritional, neurocognitive and neurodevelopmental outcomes (Ericson et al. 2018; Horta et al. 2015a; Isaacs et al. 2010).

Human milk promotes intestinal maturation, improves feed tolerance and growth (Horta et al. 2015b; Verduci et al. 2021) and helps prevent chronic conditions such as childhood and adult obesity, metabolic syndrome, cardiovascular issues and diabetes (Horta et al. 2015b; Oddy 2012; Victora et al. 2016). Providing MOM to very low birthweight (VLBW, < 1500 gm) infants is a cost‐effective intervention that supports neurodevelopment and reduces rehospitalisation due to infections (Meier et al. 2017; Mineva et al. 2023).

Direct feeding at breast (DFAB), compared to expressed maternal milk, donor human milk (DHM) or formula feeds, confers additional protection against respiratory morbidity including childhood asthma, promotes optimal craniofacial development of infants and reduces the risk of malocclusion (Klopp et al. 2017; Pérez‐Escamilla et al. 2023). Maternal skin flora from the surface of nipple, areola and breast, and infant's oral microbiota may contribute to intestinal microbiome optimisation (Granger et al. 2021). The practice of kangaroo mother care (KMC) and skin‐to‐skin contact (SSC) during DFAB enhances physiological stability, temperature control and infant metabolism (Bonet et al. 2015; Castanys‐Muñoz et al. 2016; Latuga et al. 2014; Pérez‐Escamilla et al. 2023). DFAB ensures the infant receives essential amino acids and proteins from foremilk (Pérez‐Escamilla et al. 2023; Pinchevski‐Kadir et al. 2017) and may improve their self‐regulation of energy intake, consequently protecting against obesity (Pérez‐Escamilla et al. 2023).

Therefore, based on evidence of its short and long‐term benefits, DFAB is the optimal nutritional goal for VLBW infants. However, transitioning preterm infants to exclusive DFAB is challenging. Factors such as delayed lactation, diminishing milk volume, neurological and developmental immaturity and various maternal and infant clinical factors as well as co‐morbidities can complicate this process (Bonet et al. 2015). The duration of breastfeeding is important for maximising benefits to VLBW infants as the beneficial effects of HM are dose dependent (Verduci et al. 2019). The World Health Organisation (WHO) and United Nations Children's Fund (2003) recommends 6 months of exclusive breastfeeding and continued breastfeeding alongside solid foods for 2 years or more. However, there are significant national and international variations in the breastfeeding rates of very preterm infants (Maastrup et al. 2014) with low exclusive rates globally (Huang et al. 2023; Hilditch et al. 2019; Rodrigues et al. 2018; Bonet et al. 2011). Exclusive breastfeeding rates at discharge for VLBW infants are lower and of shorter duration compared to term infants (Mitha et al. 2019). Wilson et al. (2018) reported direct breastfeeding rates ranging from 16% to 93% at discharge across 19 European regions. Maternal and infant clinical and sociodemographic factors may account for these differences, but they may also indicate insufficient targeted breastfeeding support for women. Both DFAB and exclusive maternal milk feedings at the time of discharge are linked to longer duration of breastfeeding for preterm infants (Pinchevski‐Kadir et al. 2017).

In Ireland, most VLBW infants receive expressed human milk, however few are discharged exclusively on MOM and even less directly breastfed (Philip et al. 2023; Philip et al. 2015). The variability in MOM and DFAB rates at discharge highlights the need for an in‐depth exploration into the complexities of breastfeeding at the unit level (Mitha et al. 2019). Further research is needed to inform and develop strategies that support continued feeding with MOM and direct breastfeeding (Rodrigues et al. 2018; Verduci et al. 2019, 2021; Mitha et al. 2019). Few studies have explored parents' experiences of breastfeeding VLBW infants in the Neonatal Intensive Care Unit (NICU) and following discharge to their community from a qualitative perspective. Existing research often focuses on preterm infants broadly, which may not capture the unique experiences of parents of VLBW infants (Stevens et al. 2014). Their babies typically face prolonged NICU stays, multiple interventions and a higher risk of complications, all factors that influence parents' ability to sustain milk expression and transition to DFAB.

International studies examining long‐term milk expression and direct breastfeeding for VLBW infants indicate that cultural context influences these experiences, with findings often specific to the study setting (Parker et al. 2018; Madiba et al. 2023; McLeish et al. 2024).

Some research explores parent's experience generally, focuses only on mothers, or examines isolated aspects such as milk expression (Hurst et al. 2013). Most studies consider either the NICU period (Hurst et al. 2013; Parker et al. 2018) or post‐discharge in isolation (Madiba et al. 2023). In contrast, this study explores parents' experiences across the continuum from hospital to home. A recent qualitative systematic review recommends further research on breastfeeding experiences of mothers of preterm infants across diverse contexts, including home and community environments (Srichalerm et al. 2025).

Understanding these experiences can provide valuable insights to inform national and international evidence to increase the duration of breastfeeding for VLBW infants (Mӧrelius et al. 2020). This study aimed to explore the experiences of parents of VLBW infants in sustaining long‐term breastmilk expression, their perspectives and experiences of DFAB, and their recommendations for supporting these practices in hospital and community settings to inform the development of a care pathway for DFAB.

## 2 Methods

### 2.1 Research Design

A qualitative descriptive design, as outlined by Sandelowski (2010) and Bradshaw et al. (2017), was employed using semi‐structured interviews to address the aim of the study. This study adheres to the consolidated criteria for reporting qualitative research checklist, COREQ (Tong et al. 2007) (Supporting Information S1: File 1). This research took place in a regional, level two neonatal unit, catering for an in‐house birth rate of over 4000 annually and with over 900 yearly neonatal admissions to the 19‐cot facility comprising of intensive, high‐dependency, and special care areas at the University Maternity Hospital Limerick.

#### 2.1.1 Sampling, Recruitment, and Access

Parents of VLBW infants registered in the Vermont Oxford Network (VON) database, which collects information on infants born ≤ 1500 g, or a gestational age ≤ 29 weeks and 6 days were contacted by a gatekeeper and invited to participate in the study. This gestational age threshold reflects VON's inclusion criteria for VLBW and extremely preterm infants, ensuring consistency with international benchmarking standards. Purposeful sampling ensured parents with diverse demographic backgrounds and experiences were recruited.

Inclusion criteria required participants to have a VLBW infant discharged receiving breastmilk. The inclusion of the partner acknowledges their key role in breastfeeding (Alberdi et al. 2018). Parents of infants (*n* = 22) born between 1 January 2021 and 30 June 2022, meeting the inclusion criteria were invited to participate. Eligible participants could register their interest via stamped addressed envelope or text/email to the principal investigator. They then received an information leaflet and consent form, with 2 weeks to decide on participation.

Nine women and four partners expressed interest in participating. Recruitment occurred from June 2022 to June 2023 and ceased after 12 interviews, guided by the concept of information power (Malterud et al. 2016; Braun and Clarke 2021, 2022). This approach considers factors such as study aim, sample specificity and quality of dialogue. Our purposive sample included participants with direct experience of sustaining breastmilk expression and some with experience of transitioning to DFAB. These participants provided in‐depth, relevant and detailed accounts that addressed the study aim. We concluded that a sample size of 12 offered sufficient information power for meaningful analysis. Nine mothers and three partners were interviewed. One partner was unable to attend the interview due to work commitments. The final sample included parents of three sets of twins. Participant characteristics are detailed in Table 1.

**Table 1:** Demographic details.

Variable	
Length of stay in NICU (range)	31 days to 130 days
Infants' gestation at birth (range)	26–29 weeks
Infant's weight at birth (range)	560–1495 g
Gravidity (Gr) and parity (P)	
Primigravida	2
Gr 2, P2	4
G3, P3	2
G4, P4	1
Maternal age	
20–24	1
30–35	2
35–39	5
40–44	1
Paternal age	
30–34	1
35–39	1
40–44	1
Ethnicity	
Irish Caucasian	11
Asian	1

#### 2.1.2 Interviews

Interviews were conducted using an interview schedule derived from the literature and team discussions (Supporting Information S1: File 2). Participants were offered a choice of interview modalities (in‐person, telephone or Microsoft Teams video call), enabling them to exercise agency within the research relationship and select the option that best suited their circumstances including childcaring responsibilities. To accommodate these preferences and practical constraints (e.g., childcare, travel, comfort) interviews were conducted by phone (*n* = 11) or in person (*n* = 1).

Formats varied and included seven individual interviews with mothers, one interview with both parents separately, one in‐person interview with both parents together, and one separate interview with a partner. Participants had the opportunity to ask questions and reflect on participation before giving written consent. All interviews were digitally recorded with participants' consent, and field notes were completed during and after the interviews. Interviews lasted between 29 min and 1 h 26 min and were conducted by the lead researcher (MN), an academic not involved in the provision of clinical care and who had experience in conducting qualitative interviews.

#### 2.1.3 Analysis

Interviews were transcribed in full, including participants' own words to preserve detail for interpretative analysis, anonymised, checked for completeness and managed in NVivo to organise data and support analysis. Reflexive thematic analysis, guided by Braun and Clarke's (2022) framework, involved repeated reading of the dataset and noting early analytic observations, followed by flexible, inductive coding to capture data meanings alongside reflexive notetaking. Themes were developed through an iterative, interpretive process of constructing patterns of shared meaning across the dataset conveying parents' experiences of expressing and breastfeeding their VLBW infant. Theme refinement involved returning to codes and the dataset to ensure conceptual coherence. Researchers' subjectivity and interpretations were central to coding and theme development.

The process was underpinned by methodological consistency and reflexion. Themes were developed collaboratively through discussion and exploration and consideration of alternative interpretations, acknowledging that knowledge is partial and context‐based. To support transferability, we provide detailed narrative accounts along with descriptions of sampling and participant demographics.

### 2.2 Ethical Considerations

The study was conducted in accordance with the Declaration of Helsinki (World Medical Association 2013) and approved by the Research Ethics Committee (REF 130/9). The general data protection regulation (GDPR) compliance for research by the Health Service Executive (HSE) was ensured and all study data was stored on GDPR secure cloud storage (Philip 2019).

### 2.3 Reflexivity

Three researchers (MN, JMC, SH) conducted data analysis, one was a practicing midwife not based in a neonatal unit, and two were midwifery lecturers with neonatal experience. Reflexive dialogue took place during weekly meetings to critically question assumptions, positionality, and how this influenced interpretation. A guiding question was: Are we being influenced by our own experience or does this theme genuinely reflect participants' accounts? This ongoing reflexivity helped ensure interpretations were meaningfully connected to participants' accounts. Final themes were critically reviewed by the wider research team, comprising both clinicians and academics.

### 2.4 Ethics Statement

Ethical approval was obtained from the Mid‐West Regional Ethics Committee in November 2019 (REF 130/9).

## 3 Results

Twelve parents (mothers = 9, fathers = 3) participated in the study. Interviews took place during the COVID‐19 pandemic, and all parents mentioned pandemic‐related community‐level and hospital‐level restrictions that impacted their experience. Participant and infant demographics are presented in Table 1.

### 3.1 Theme 1: Sustaining Breastmilk Expression for Preterm Infants

**Figure 1:**
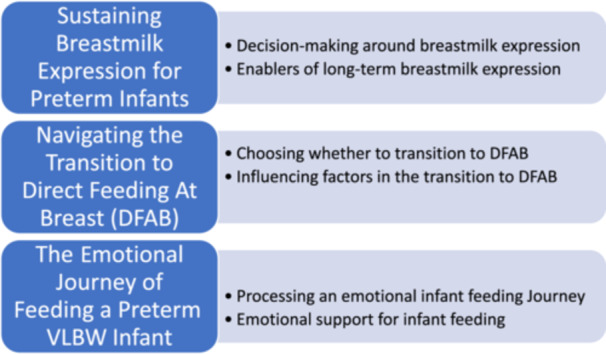
Themes and subthemes.

In this theme, parents shared how they made decisions about breastmilk expression and what helped them continue long‐term Figure 1.

#### 3.1.1 Decision‐Making Around Breastmilk Expression

Many parents' conversations with healthcare professionals (HCPs) affirmed or influenced their decision to express breastmilk. Interactions were most effective when they were non‐judgemental, personalised, conveyed with empathy and provided realistic expectations about expressing for a VLBW infant.

One participant recalled how a consultant's encouragement and choice of terminology instilled confidence in her ability to provide milk for her infant:

But he just was such a kind man (consultant) and very, very nice about it, just literally going, don't put any pressure on yourself, every drop counts, liquid gold, he says, you know, he was so nice, he inspired trust and confidence without it being forced on us.

(P2)

The inclusion of partners in these conversations had the potential to influence their commitment to breastfeeding.

When I was pregnant, he (partner) did not want me to breastfeed and then as soon as he was born, and they said he needs breastmilk then that flipped the switch. And he totally was all about it. He was washing my pump parts. He was like, the best support ever.

(P5)

However, one partner felt excluded, perceiving that discussions were directed solely at the mother, highlighting the importance of ensuring both parents feel involved.

It wasn't a kind of conversation for both of us, it was more directed at my partner, and I just stood there listening.

(Partner, 10)

Most participants were satisfied with the length of time they were able to sustain milk expression. For some, the decision to discontinue expressing was pragmatic, made in response to a reduction in milk supply and the transition home:

I would have liked to have kept going. I think, well I'm not sure, I feel if he had latched, I would still be breastfeeding. I wanted to do it for the year. That was my goal, but then when the supply dropped in the summer when we came home, I ended up finishing.

(P5)

#### 3.1.2 Enablers of Long‐Term Breastmilk Expression

Women identified a range of supports that facilitated sustained milk expression including written, verbal and practical guidance on hand and pump expression. They expressed a preference for in‐person consultations as these provided an opportunity for real‐time, personalised discussions to address their concerns. As one participant explained, in‐person support helped ‘*clarify everything…the question in your head*’ (P8), which they felt could not be achieved through written materials or remote consultations: ‘*because it's very practical, like they kind of have to show you, they nearly have to look at you doing it*’ (P3). Another participant noted:

I think that if you're talking to someone you can actually ask, well this has happened to me, or well what if this happens? You know, you can't do that with a leaflet. I think a conversation is better for me.

(P5)

The provision of a hospital‐grade electric pump was instrumental in supporting mothers to continue milk expression. However, parents were often hesitant to ask busy HCPs for bottles and labels, suggesting that these resources be readily accessible.

Now neonatal are brilliant, and they're absolutely up the walls. we're asking them constantly for pump, packs, you feel like you're bothering them when they have so much to do.

(P6)

The dedicated expression room in the NICU provided a break from the intensity of the unit and offered opportunities to connect with other parents. Participants also expressed a desire for more opportunities to express milk beside the incubator, or during SSC:

One thing that I would ask them to change, to put the pumps beside the cubicle, or beside the incubator, make it the norm.

(P6)

Participants highlighted the need for more freezer storage space for expressed breast milk and consistent guidance on using fresh and frozen breast milk. As one parent noted, ‘*loads of it just ended up in my freezer’* (P1).

Support from neonatal staff and lactation consultants (LCs) helped women sustain long‐term milk expression. Participants valued scheduled weekly check‐ins, as it was sometimes only in hindsight that they realised they needed further guidance, as illustrated by the following quotation

I was pumping for an hour. They didn't see a problem with me because I was bringing them loads of milk.

(P6)

Those struggling with expression preferred private consultations with a LC or nurse/midwife and saw the need for a dedicated LC for the NICU.

That day everyone in the room wanted to talk to her, (the LC) the poor woman was in demand.

(P9)

In the community, participants reported variable access to specialist lactation support. Those who did have access valued the involvement of experienced PHNs and LCs who organised hospital‐grade pumps, maintained regular phone contact, and provided support following discharge.

The PHN has been an absolute great support here in the community, she has touched base with me, I don't know how many times even about the expressing.

(P2)

Participants described several challenges that made sustained expression difficult. These included personal and lifestyle factors such as caring responsibilities for other children, day‐to‐day demands, and travel to and from the neonatal unit. One participant highlighted the lack of designated pumping areas in community settings. Other challenges included insufficient information and education on expressing practices, separation from babies, caring for twins, reduction in milk supply before or after discharge, and limited family support.

### 3.2 Theme 2: Navigating the Transition to DFAB

This theme explores the complex decision‐making process of transitioning from milk expression to DFAB and the range of factors that influenced this experience.

#### 3.2.1 Choosing Whether to Transition to DFAB

Participants described a range of perspectives on DFAB. Some were motivated by a desire to experience breastfeeding for the first time, while others had previously breastfed and intended to do so again. Women who experienced DFAB expressed satisfaction for various reasons:

I was able to try breastfeeding. You know, I had that experience. I didn't miss out on that.

(P3)

A few women choose not to breastfeed directly, expressing concerns about managing it at home. They felt this decision was appropriate for their individual circumstances.

It's a bit of a turmoil because you know breastmilk is best for them you know, as a mom, you're thinking oh god they could do with that but at the same time you're thinking how would I manage all this at home and because I know breastfeeding takes up so much time … I know for me it was the right decision but…

(P4)

Participants were initially informed that their premature infant would not be able to breastfeed directly. For some, this belief persisted throughout their NICU stay:

When I did try, I was kind of taken off guard that it was possible to even try.

(P7)

One woman explained that she hadn't pursued DFAB because her priority was ensuring her baby received breastmilk, regardless of the method of feeding.

I probably didn't like push for it either. Because from the point that you know, you're just so worried and you are just hoping that once he was getting the food like, through the syringe or fed by the tube or whatever, that you didn't mind once he was taking the feed.

(P2)

#### 3.2.2 Influencing Factors in the Transition to DFAB

Parents described several factors that influenced their ability to transition to DFAB.

##### 3.2.2.1 Infant‐Related Factors

A wide range of infant‐related factors impacted DFAB. Participants spoke about the challenge of establishing breastfeeding when babies experienced conditions such as tongue tie or reflux:

My little boy suffers with reflux so I add thickener to his milk…I put him on, kind of, just a little bit. But he wouldn't take a full feed.

(P1)

Some parents of twins reported that their original plans for DFAB changed to long‐term milk expression.

So it turned out very different than what I was expecting. So, it was all expressing for me rather than breastfeeding, so it didn't go to what I had planned I suppose.

(P7)

Having one twin in the hospital while the other twin was at home meant that mothers were trying to balance expressing milk with DFAB, which proved challenging for some. For other parents, getting their baby home was the priority, and establishing bottle feeding was perceived as an important step in facilitating discharge.

We had been there for so long with this little baby, we just wanted to get home, he was almost 2 months old, and we had missed out so much of the little baby bundle.

(P9)

Participants described challenges initiating breastfeeding when their infant had become accustomed to bottle feeding.

I did want to breastfeed originally, I wanted to latch him, but he was so used to the bottle then at that stage. He hadn't the interest. It was too slow for him.

(P5)

While others were concerned about determining milk transfer:

He has to be hitting the quota to get out of here … so when he was on the boob, how much was he getting?

(P9)

##### 3.2.2.2 Health System Factors

Health system factors encompass information and support from HCPs across hospital and community settings and discharge planning.

Participants appreciated that HCPs did not pressure them to breastfeed directly and spoke about the significance of ‘*keeping an open conversation’* (P4) and sensitively discussing the importance and realities of transitioning to DFAB:

I suppose they try to be neutral, but as a mother, all you want is the facts and the practicalities of it.

(P4)

Support from HCPs with DFAB was appreciated:

They (HCPs) always made it good and they had screens for privacy and they took time to help if I needed it and so I have to say they were very nice and helpful like that.

(P7)

Participants were often reluctant to ask for help, aware of how busy NICU staff were and wanting the focus to remain on their infant's care. They suggested that a structured DFAB plan may have better supported their transition to direct breastfeeding.

As I say I didn't end up breastfeeding but maybe if there was a set plan.

(P7)

The LC was assigned to the hospital and attended the NICU on request. However, participants felt that having a LC based on site would have provided more consistent and accessible support:

I cannot fault the unit, and they were absolutely super, everyone was fantastic. But I do think that if there was somebody full time just dedicated for women with breastfeeding.

(P2)

Some participants successfully initiated DFAB at home, while others were unable to get their baby to latch even with assistance from a community‐based LC:

And I brought him home a few weeks later. And we tried to put him on. And she (Community based LC) felt then that it was gone too far. And we did have this conversation, that if there was somebody just there, just dedicated to try at an earlier stage that it might be a better approach.

(P2)

Parents expressed a desire for more advanced notice of discharge to better plan for the initiation and continuation of DFAB.

Oh, definitely, the opportunity to stay in the parents' room was there. But the timeline just snuck up on us.

(P5)

##### 3.2.2.3 Partner Influences

Some partners conveyed support for the woman's decision while others expressed concerns about the practicalities of managing breastfeeding alongside caring for a newborn and other children.

He was always supportive to what I was going to do. Like, he knew that I really wanted to get him onto the breast. And he was always encouraging.

(P2)

I kind of did want to breastfeed but I remember having a conversation with my husband and he was like; but sure X we have two and we have a x year old, and you know, you're not gonna be able to manage and obviously, you know, I was a bit like “ah sugar”.

(P4)

### 3.3 Theme: The Emotional Journey of Feeding a Preterm VLBW Infant

This theme captures parents' emotional experiences and support systems in sustaining expression and transitioning to DFAB.

#### 3.3.1 Processing an Emotional Infant Feeding Journey

Participants described a range of fluctuating emotional responses while learning to express breastmilk, including shock and grief related to preterm birth and uncertainty about their infant's condition.

At times when he got sick, when he was really unwell, and you weren't expecting it. It's kind of hard you know; you are expected to come home and keep going at the same time.

(P1)

Participants valued the opportunity to express for their preterm infant seeing it as their unique contribution to their infant's health:

It's probably the only thing that you could do for him. Yeah. So that's, that keeps you going.

(P1)

Expressing breast milk became the woman's focus described as ‘*just my job’* (P1) which served as a distraction from the reality of having their infant in the NICU, provided a “*sense of purpose*” (P2), and helped women gain control over a situation they felt they had no control over.

The expressing, that was massive for my mental health and who would have thought it. So that, to be honest, I was mentally not too bad while they were in hospital.

(P4)

However, the emotional cost of long‐term expressing was also evident. Some mothers described sacrificing time with their infant to maintain their expressing schedule while others spoke of the loneliness of nighttime pumping.

Sometimes I feel like I sacrificed my time with the baby to pump. That sounds awful but…

(P1)

Sitting in the kitchen on your own pumping and he's upstairs in bed having to go to work the next morning, it's, it's really lonely, pumping.

(P6)

It was only when their infant was home, and mothers had time to reflect that the emotional toll of long‐term expressing and its impact on their mental well‐being became fully apparent for some participants.

I don't think you realise a lot of times when you're there, you're in the bubble I suppose. I don't know how we did it really.

(P1)

They also spoke of a seesaw of emotions when deciding not to pursue DFAB and to stop expressing ranging from relief at no longer being consumed with milk expression to regret and self‐blame for being unable to continue:

It didn't work that time, but maybe if I had stuck with it or there was a plan in place, eventually it would have.

(P7)

Weaning from expressing or DFAB was described as a time of heightened emotional vulnerability.

I did find when I stopped breastfeeding and expressing, emotionally like hormones. Emotionally I did feel I started to get worse then actually, I felt you know that that's when I really started to dwell on it.

(P3)

#### 3.3.2 Emotional Support for Infant Feeding

Emotional support played a critical role in sustaining long‐term breastmilk expression and, for some, transitioning to DFAB. Support from HCPs was especially appreciated when it was consistent, encouraging and non‐judgemental.

I think just being seen and encouraged, as silly as it sounds. That's the best thing you could do and ‘how is your supply?’, and you know.

(P1)

Parents appreciated a range of supportive behaviours including regular updates, making a ‘*bit of small talk*’ (Partner 2), and offering ‘*practical advice, but nothing too pressured*’ (P3).

They were really, really encouraging and really non‐judgmental the nurses and midwives in the NICU. They would make you feel you were the best thing ever.

(P3)

Participants had various views on connecting with other parents. Some found comfort in shared feeding experiences while others preferred to keep to themselves due to high emotions:

A safe space (milk room) where everyone chatted and kind of went, ‘What's your story? a lot of it was a support in itself just to chat to other moms in there.

(P3)

I suppose everybody's situation is different. Emotions were very high. So sometimes you kind of keep to yourself and try to get through your own day.

(P1)

It was only when the infant was discharged that some women recognised their need for formal perinatal mental health support which they valued.

So, I have reached out, I have been getting support from perinatal mental health services.

(P3)

However, one partner highlighted the lack of psychological support available for fathers:

The thing is, with all the support and everything in the place, it is all for the woman. I struggled to find any forums or any form of support for the male side of it.

(Partner 10)

## 4 Discussion

The findings of this research enrich our understanding of parents' experience of sustaining long‐term milk expression and transitioning to DFAB. Access to equipment, SSC, practical and emotional support from partners and HCPs sustained milk expression. Infant‐related issues, lactation support from HCPs across hospital and community settings and partner involvement influenced the transition to DFAB. Strategies to optimise the duration of milk expression and increase the likelihood of transitioning to DFAB are presented in Table 2 and Supporting Information S1: File 3.

**Table 2:** Themes, subthemes, participant quotations and recommendations (Parent‐Suggested and Researcher‐Derived).

Theme	Subtheme	Quotations (examples)	Recommendations
Theme 1: Sustaining breastmilk expression for preterm infants	Decision‐making around breastmilk expression	‘Tell me the good, the bad, the ugly. Yeah, I'll make my decision. You know, you won't scare me off by telling me the truth. Just tell me the truth.’ (P2). “Because if the husband is part of this process, he is more likely to cooperate and help you out and understand that my wife is in this situation, this difficult situation, I must step in and do my part too” (P8)	Provide parents with personalised, realistic information about the importance and process of providing MOM for their preterm VLBW infant.
	Enablers of long‐term breastmilk expression (informational, instrumental, practical)	*Informational support* ‘I think It could be like simple things. Even just to acknowledge that the male partner is there. Have a chat with him the very same you would with the mother. Treat him a bit more equal in terms of our presence there that you are not a nuisance, you are not in the way’ (Partner 10) *Instrumental support (resources for milk expression)* ‘They kind of gave me everything I needed, the pump and the materials and supplied me with a community pump for going home. A breastfeeding log so I could keep track of my feeding schedule for my volume’ (P1). “Definitely there wasn't enough freezer space… there's nothing worse than obviously pumping, and I have nowhere to put my own milk” (P4) *Instrumental support (Place/venue to pump)* “That was just brilliant, expressing then while you were holding him. Definitely your supply would be better after holding him and having skin to skin with him” (9) “If they could have in that room… some partition walls or something that you could go in and you wouldn't know who was beside you, you could be on your phone or whatever little time you were in the room. Sometimes it was lovely chatting away to people but other times it wasn't” (P9). “If you could express breastmilk in the room next to the incubator, with the screen up it would be much easier way of doing it” (Partner 10). *Practical support (healthcare professionals and family)* “Again, they did tell me all that, the help was there if we wanted. I mean to be fair they did. But you would have to reach out yourself which I didn't at the time, not really like for any reason. Now they wouldn't always be there, …but I didn't discuss it really” (P3). “May be if there was someone (lactation consultant) more sliding in and out or one designated to Neo to check up on you every day or second day, you know ‘are you OK” (P9). “I can't remember who she was, but she was definitely a lactation or something in the community. And that was a huge benefit, and it was great to have that” (P7).	*Informational support recommendations* Schedule weekly sessions with parents to address their questions and concerns. Train NICU and community staff on long‐term milk expression. *Instrumental support (resources)* Continue providing hand expression kits, hospital‐grade pumps and ensure milk expression equipment is readily available in designated milk expression rooms. Ensure there are sufficient refrigerators for storing EBM in the NICU. *Instrumental support (Place to pump)* Offer a range of pumping location options including during SSC/KMC, beside the incubator, or in a designated milk expression room. Provide flexible SSC opportunities for both parents and ensure that parents of twins have opportunities for SSC contact with both infants. Ensure parent input into the design and improvement of milk expression rooms. *Practical support (HCPs and family)* Ensure parents have access to scheduled expert lactation support for long term milk expression for a preterm baby across both hospital and community settings. Appoint designated lactation consultants specifically dedicated to supporting parents in the NICU. Ensure the continuation of specialist lactation support in community settings for parents of preterm VLBW infants.
Theme 2: Navigating the transition to direct feeding at breast (DFAB)	Choosing whether to transition to DFAB	‘I was told from the start I was told they were premature there was no way I could breastfeed from the start that they would have to be that little bit older that they were being tube fed for the first while. And actually it was all of a sudden one day whatever particular nurse said do you want to try to breastfeed. I didn't realise it was possible at that stage, but she said give it a try and there was no plan as such in place just give it a go. That was kind of it’ (P7). ‘But they were very quick then to put the babies on the formula more, as soon as I had said ‘Oh, you know, when I was still in the phase of, will I, won't I… I don't know whether it's okay or not, and I wonder if they had encouraged me a bit more to try and keep with the breastfeeding, would I have done?’ (P4). ‘I was glad when she was finished with it; it took a lot of pressure off in terms of that commitment every day’ (Partner 10).	Introduce the option of DFAB early in the neonatal intensive care journey as a sustainable and achievable feeding option for preterm VLBW infants. Connect parents with other mothers who have successfully transitioned to DFAB. Maintain an open dialogue about transitioning to DFAB and revisit this decision with parents. Ensure that PHN or community‐based lactation consultant discuss the mother's feeding decisions and revisit the option of DFAB upon the baby's discharge home.
	Influencing factors in the transition to DFAB	*Infant related factors* ‘I think to be fair to people in the hospital, because obviously, they're so busy, they want to get the girls feeding and discharged, you know, which is perfectly normal, both sides want to get to keep the babies healthy and big enough to go home’ (P3). ‘But I had always been a bit sceptical about how much the kids would be getting, because obviously you don't know, your supply, you don't know. You know, you don't know the numbers. And that would drive me crazy’ (P6) *Health system factors* ‘When the girls were discharged a LC, did call to me a few times. She was helpful and showed me kind of, you know, best ways to breastfeed really, and to be fair, I did especially with the smaller twin, I did breastfeed her for the first few weeks nearly exclusively’ (P3) ‘I didn't get a visit from the lactation consultant into my home, but in fairness she did ring once or twice, definitely to see how I was’ (P4). ‘My public health nurse was actually a lactation consultant as well. So, they were telling me you know she would support you and to bring her out and she was, she was great. But he just couldn't take to it, we tried, you know, the tube as well and he was actually getting it from the bottle but on the breast kind of thing. But no, it just didn't suit him’ (P5)	Co‐design national and local DFAB guidelines with input from HCPs and parents to ensure relevance. Develop and implement training programmes for hospital and community‐based staff to support women with preterm infants in sustaining long‐term milk expression and transitioning to DFAB. Collaboratively develop a personalised DFAB transition plan with parents outlining realistic and achievable milestones for their baby's progression in both hospital and community settings. Ensure parents have access to scheduled expert lactation support in both hospital and community settings to promote DFAB. Provide alternative feeding options to bottle feeding for VP infants in the NICU. Offer opportunities for women to stay in the NICU prior to their baby's discharge to support the transition to DFAB.
Theme 3: The emotional journey of feeding a preterm VLBW infant	Subtheme 1: processing an emotional infant feeding Journey	‘At times when he got sick that I had to, when he was really unwell and you weren't expecting it. It's kind of hard you know, you are expected to come home and keep going at the same time. At the same time, it's probably the only thing that you could do for him. Yeah. Yeah. So that's, that keeps you going’. (P1) ‘It was a massive focus (expressing breast milk). And it actually gave me a bit of sense of purpose in the first, especially the first while when it was very traumatic’ (P2) ‘The fact that I could (express), It probably helped us both, you know, in that regard. Just that something was coming from our side’ (P3).	Inform parents about the range of emotional responses they may experience following a preterm birth, during long‐term milk expression and when stopping expression and breastfeeding. Regularly screen parents who have experienced the birth of a preterm VLBW infant for perinatal mental health symptoms in both NICU and community settings, using validated tools.
	Sub theme 2 emotional support for infant feeding	‘PHN put me back in contact with the perinatal support. I wouldn't like to say I wasn't open to going myself, it was never that I said that I don't need it. But I do think the public health nurse was great. And then the GP to be fair, she's actually very supportive as well’ (P3). ‘There's nothing, there is absolutely nothing. And I've met a few people and they said that their partners found it really hard as well. That they could have done with having someone to talk to, someone in the same boat’ (P6).	Provide information to parents about perinatal mental health supports in both hospital and community settings. Implement training programmes that support parents of VLBW infants adapt to their parental role, with specific guidance for parents of multiples.

Abbreviations: DFAB, Direct Feeding At Breast; EBM, Expressed Breast Milk; HCPs, Healthcare Professionals; KMC, Kangaroo Mother Care; LC, Lactation consultant; MOM, Mothers own milk; NICU, Neonatal Intensive Care Unit; PHN, Public Health Nurse; SSC, Skin to Skin Contact; VLBW, Very Low Birth Weight; VP, Very Preterm.

Consistent with previous research (Mӧrelius et al. 2020), mothers valued expressing breastmilk as a meaningful contribution to their infant's health. Our findings suggest that HCP‐led conversations, involving both parents, outlining the importance of HM and providing realistic expectations about long‐term expressing, may foster and strengthen parental commitment to breastfeeding VLBW infants. The pivotal role of MOM in preterm care and its contribution to healthy outcomes warrants promotion and publicity utilising social media channels, a resource which many young parents seek information from during pregnancy and postpartum (Morse and Brown 2022).

Ongoing encouragement from HCPs, partners and peers motivated mothers to sustain milk expression, echoing findings from international research (Flacking et al. 2021; Huang et al. 2023; Matriano et al. 2022). Despite this, some participants in our study reported discontinuing breastmilk expression earlier than planned for a variety of reasons. This highlights the importance of ongoing, sensitive, and scheduled assessments and conversations about the value of HM which may help inform parents' expectations and extend the duration of milk expression. Mothers of twins found long‐term expression challenging, a finding supported by Wilson et al. (2018) who emphasised the need for targeted support for this cohort.

Male partners felt they had limited influence over the decision to initiate breastfeeding, yet they played a key role by offering practical and emotional support. The importance of partner involvement is corroborated by Earle and Hadley's (2018) review. Inclusive conversations may help partners feel more involved in the decision‐making process, while simultaneously securing their support for the decision. Including partners in breastfeeding interventions has been shown to increase breastfeeding rates (Abbass‐Dick et al. 2019; Mahesh et al. 2018).

Consistent with previous findings, participants emphasised the importance of easy access to milk expression equipment (Flacking et al. 2021; van Veenendaal et al. 2022). Mothers valued expressing beside the incubator or during KMC/SSC, and parents desired more privacy and flexible uninterrupted SSC. These options may help minimise the conflict between spending time with their baby and adhering to frequent milk expression schedules (British Association of Perinatal Medicine 2022). Supportive neonatal unit design that facilitates KMC and SSC has been associated with increased milk supply and responsive feeding (Alves et al. 2013; Flacking et al. 2021; O'Callaghan et al. 2019; World Health Organisation 2022).

Throughout the data, participants described aspects of high‐quality care that supported their breastfeeding journey. These included personalised, empathetic, non‐judgemental interactions free from pressure, indicating that women valued respectful, person‐centred breastfeeding support, a view reflected in the literature (Nardella et al. 2025; Schmied et al. 2011). Schmied et al. (2011) found that women experience breastfeeding support along a continuum from authentic presence to disconnected encounters and highlighted the importance of a facilitative approach that encourages dialogue, provides realistic information and supports parental autonomy. Our findings echo this, as participants expressed a desire for honest, balanced information about expressing and DFAB, including potential challenges, rather than idealised messages that do not reflect reality.

Challenges with initiating and sustaining DFAB are well documented (Hilditch et al. 2019) and were echoed in our findings. Multilevel support across NICU and community settings is needed to help VLBW infants transition to DFAB. Earlier, realistic discussions may help parents consider DFAB as a viable option. Parents often perceived that establishing bottle feeding would expedite discharge, influencing their feeding decisions. However, evidence suggests that avoiding or minimising bottle use during breastfeeding establishment may increase the likelihood of full or partial breastfeeding without delaying discharge (Allen et al. 2021). HCPs need to discuss with parents the implications of DFAB on discharge timing and breastfeeding duration, while promoting it as the optimal, sustainable option.

Parents are often hesitant to seek breastfeeding support, fearing they might burden busy NICU staff and distract from their infant's care. This highlights the need for scheduled personalised informational, practical and emotional support from trained HCPs. Proactive lactation support is associated with improved HM feeding outcomes (Fang et al. 2021; Flacking et al. 2021; Hilditch et al. 2019; Hoban et al. 2022). The importance of specialist lactation support facilitated by a neonatal infant feeding lead or LC based in the NICU is widely recognised (British Association of Perinatal Medicine 2022; Chetwynd et al. 2019; Gharib et al. 2017; Hoban et al. 2022). However, it is important that all NICU nurses and midwives have the knowledge and skills to support DFAB, as LCs are not typically available 24 h a day in most units. Over reliance of LC support may lead to gaps in care and missed opportunities to support parents effectively.

Ongoing specialist lactation support in the community is essential. PHNs and community‐based LCs must be equipped with the necessary knowledge and skills to support parents of VLBW infants, as DFAB may not be initiated or fully established during hospitalisation (Flacking et al. 2021). Strengthening cross‐setting integration can help families build supportive relationships with PHNs from admission through to discharge and beyond (British Association of Perinatal Medicine 2022).

Our study highlights the emotional toll that long‐term milk expression and prolonged NICU stays exert on parents' perinatal mental wellbeing. Within this context, mothers faced complex decisions about continuing expression and transitioning to DFAB. Elevated stress, anxiety and depression (Nguyen et al. 2023; Pace et al. 2016) can hinder transitions to parenthood, breastmilk production, decision‐making, the sustainment of milk expression and the success of DFAB (Flacking et al. 2021; Schwab et al. 2024). Schwab et al. (2024) argue that lactation support must address both psychological and physiological needs. Thomson et al. (2022) reported varied intervention efficacy and a lack of focus on fathers' psychological needs, a gap identified by one partner in our study. Peer support may help build parental confidence and emotional resilience (Hynan and Hall 2015). Integrating personalised emotional support into a DFAB care pathway may enhance parental perinatal wellbeing.

Recommendations from our study, outlined in Table 2, align with Ireland's National Standards for Infant Feeding in Maternity Services (Health Service Executive 2022), which incorporate the WHO/UNICEF 10 Steps under the Baby Friendly Hospital Initiative (BFHI). While these standards reference neonatal care, our findings highlight the need for more detailed, neonatal‐specific guidance. This is consistent with international evidence, including Maastrup et al. (2022) Neo‐BFHI framework, which adapts the 10 Steps for neonatal wards and emphasises practices such as evidence‐based breastfeeding training for NICU staff, continuous SSC, early initiation and maintenance of lactation, use of breastfeeding‐supportive feeding methods, collaborative transition strategies, avoidance of bottles until breastfeeding is established, and continuity of support after discharge. Finally, co‐producing DFAB guidelines and quality improvement resources for hospital and community settings can help ensure they are developed cognisant of parents' preferences, needs and values (Flacking et al. 2021).

### 4.1 Study Strengths and Limitations

This is a small‐scale study, which included parents from different sociodemographic backgrounds from one regional NICU. While the study was conducted with parents of VLBW infants in Ireland, the findings are likely to be relevant to similar populations in high‐income countries with comparable neonatal care systems. However, cultural and healthcare system differences may influence the extent to which these findings apply in other contexts. Offering participants a choice of interview modality supported engagement and inclusivity by reducing barriers such as childcare and travel. However, variation in interview modes may have influenced the interaction and depth of responses, despite efforts to maintain consistency through the same interviewer and interview schedule. Despite extensive recruitment efforts, only three fathers volunteered to participate in the study. While this limited representation did not allow for a separate, in‐depth analysis of partners' perspectives, their views were integrated within the overall thematic analysis to provide additional context and enrich understanding of parental experiences. Further research is needed to explore partners' experiences of supporting nature's norm of DFAB. As the study was conducted during the COVID‐19 pandemic and the resultant access limitations for parents, some of the perceptions could have been influenced by the lockdown/stringent public health measures.

### 4.2 Patient and Public Involvement (PPI)

Principles of PPI in research were embedded from the outset guided by the HSE Research and Development framework (Health Service Executive 2025). These principles emphasise partnership and collaboration, respect and transparency, inclusion and equity, empowerment and shared decision‐making, trust, flexibility, impactful involvement, and patient and public guided research priorities.

In this study, representatives from a registered neonatal charity (Registration number 20073732), including a mother of a former extremely low birthweight infant, who provided MOM and progressed to DFAB, were consulted during project development. Their engagement aligned with core PPI principles and included reviewing the interview schedule, proposals for the dissemination of findings, and guidance on the value of partner involvement among the parental cohort. Their involvement helped ensure the study was relevant and sensitive to the needs of families.

## 5 Conclusion

The provision of MOM is one of the most evidence‐based and cost‐effective interventions available for optimising the health of VLBW infants. Our findings suggest that a combination of multilevel, varied, and flexible support strategies adapted to parents' diverse needs are required to increase MOM feeding at discharge and facilitate DFAB. A structured plan with scheduled sensitive consultations from trained HCPs is needed to support women to transition to DFAB. Emotional and psychological support offered to parents during the prolonged and challenging neonatal journey after the birth of preterm infants is paramount to foster the parent‐infant bonding that facilitates the success and sustenance of DFAB.

## Author Contributions

The work was jointly conceived in discussion with all authors. Interviews were conducted by Maria Noonan. Thematic analysis was undertaken by Maria Noonan, Jan McCarthy and Sandra Healy. Themes and subthemes were discussed and revised by the whole team. Maria Noonan and Roy Philips wrote the first draft of the article and edited the final version, and all contributed to subsequent reviews. All authors approved the final version of the paper.

## Conflicts of Interest

The authors declare no conflicts of interest.

## Supporting information

Supporting File 1

Supporting File 2

Supporting File 3

## Data Availability

The data that supports the findings of this study are available in the supporting material of this article.
