# Supplementary material for: Sustaining Breastmilk Expression and Direct Feeding at Breast for Very Low Birth Infants: A Qualitative Exploration of Parental Perspectives
Source: Matern Child Nutr. 2026 May 27;22(3):e70193. doi: 10.1111/mcn.70193 (PMC13214513; doi:10.1111/mcn.70193)
Supplement: Supplementary file 2 — Supporting File 2 [file MCN-22-e70193-s002.docx]

**Supplementary File 1: Interview Schedule**

| **Interview schedule (mother)** |
| --- |
| 1. Tell me about your experience of breastfeeding your baby in the NICU? 2. What helped you to express breast milk for your baby? 3. What facilitated you to exclusively breastfeed your baby? 4. If exclusive breastfeeding was not possible, can you identify what affected exclusive breastfeeding?      1. What structures would support you to continue expressing/breastfeeding in the community? |
| **Interview schedule (partner)** |
| 1. Tell me about your experience of receiving breastfeeding support in the NICU? 2. What facilitated you to support your partner to express breast milk for your baby? 3. What facilitated you to support your partner to exclusively breastfeed your baby? 4. If exclusive breastfeeding was not possible can you identify what affected exclusive breastfeeding? 5. What support structures would support you and your partner to continue expressing/breastfeeding in the community? |
