# Supplementary material for: Sustaining Breastmilk Expression and Direct Feeding at Breast for Very Low Birth Infants: A Qualitative Exploration of Parental Perspectives
Source: Matern Child Nutr. 2026 May 27;22(3):e70193. doi: 10.1111/mcn.70193 (PMC13214513; doi:10.1111/mcn.70193)
Supplement: Supplementary file 3 — Supporting File 3 [file MCN-22-e70193-s003.docx]

**Supplementary file 3: Themes, subthemes, participant quotations and recommendations (Parent-Suggested and Researcher-Derived).**

| **Theme** | **Subtheme** | **Quotations (examples)** | **Recommendations** |
| --- | --- | --- | --- |
| ***Theme 1: Sustaining Breastmilk Expression for Preterm Infants*** | ***Decision-making around breastmilk expression*** | “Tell me the good, the bad, the ugly. Yeah, I'll make my decision. You know, you won't scare me off by telling me the truth. Just tell me the truth. You know, don’t build it up like, it's this magically magical experience, and there's such a bond and it's great for your baby. And then you're struggling at home, pumping away using the machine that you don't know how to use and you're just bawling crying” (P2).  “I didn't know that it would be as gruelling as it was” (P6).  “He needed to be part of that conversation as much as me when doctors come up, like the dads were actually included as well, you know, because some people you'd meet they would just be looking at the mom and talking to the mom. But I did really find that they were really including the two of us” (P3)  “Because if the husband is part of this process, he is more likely to cooperate and help you out and understand that my wife is in this situation, this difficult situation, I must step in and do my part too”” (P8)  “That was in my head every little bit helped him … after what the consultant said your milk is gold, I said here I better give this a good go then” (P9). | Provide parents with personalised, realistic information about the importance and process of providing MOM for their preterm VLBW infant.  Provide personalised information to support parents in making an informed decision about when and how to wean from expressing breast milk. |
|  | ***Enablers of Long-term Breastmilk Expression (informational, instrumental, practical)*** | ***Informational support***  “I think that if you're talking to someone you can actually ask, well this has happened to me, or well what if this happens? You know, you can't do that with a leaflet. I think a conversation is better for me” (P5).  “I think It could be like simple things. Even just to acknowledge that the male partner is there. Have a chat with him the very same you would with the mother. Treat him a bit more equal in terms of our presence there that you are not a nuisance, or you are not in the way” (Partner 10)  **Instrumental support (resources for milk expression)**  “They were really, really supportive, the lactation consultants. I think it was two different maybe that I met on a couple of occasions and they kind of gave me everything I needed, the pump and the materials and supplied me with a community pump for going home. A breastfeeding log so I could keep track of my feeding schedule for my volume” (P1).  “When I eventually got down to the unit, I have to say they were fantastic with giving me, making sure I had kits and bottles and labels” (P2)  “Definitely there wasn't enough freezer space. I do, do think a backup freezer somewhere would have probably been handy for those times when there's a lot of babies and a lot of women pumping because there's nothing worse than obviously pumping, and I have nowhere to put my own milk” (P4)  **Instrumental support (Place/venue to pump)**  “That was just brilliant, expressing then while you were holding him. Definitely your supply would be better after holding him and having skin to skin with him” (9)  “If they could have in that room… some partition walls or something that you could go in and you wouldn’t know who was beside you, you could be on your phone or whatever little time you were in the room. Sometimes it was lovely chatting away to people but other times it wasn’t “(P9).  “If you could express breastmilk in the room next to the incubator, with the screen up it would be much easier way of doing it” (Partner 10).  **Practical support (healthcare professionals and family)**  “The lactation consultants are always around, or they can call for them to come down” (P1)  “Again, they did tell me all that, the help was there if we wanted. I mean to be fair they did. But you would have to reach out yourself which I didn't at the time, not really like for any reason. Now they wouldn't always be there, …but I didn't discuss it really” (P3).  “I think maybe the lactation consultant should come around, maybe a bit more” (P6).  “May be if there was someone (lactation consultant) more sliding in and out or one designated to Neo to check up on you every day or second day, you know ‘are you OK” (P9).  “I can’t remember who she was, but she was definitely a lactation or something in the community. And that was a huge benefit, and it was great to have that” (P7).  “To be honest he (partner) was great when I got home expressing at three in the morning and he would get up and run off to the fridge with the milk, go and wash the stuff and sterilize it. He was great. He knew it had to be done and he knew it had to be done cleanly and he would go off and do his little bit too” (P9) | ***Informational support recommendations***  Schedule weekly sessions with parents to address their questions and concerns.  Train NICU and community staff on long-term milk expression, including the use of both freshly expressed and frozen breast milk, to ensure consistent information is provided to parents.  Provide information resources highlighting the role of partners and families in supporting long-term milk expression.  ***Instrumental support (resources for milk expression recommendations)***  Continue providing hand expression kits, hospital-grade pumps and pumping supplies to support long-term milk expression.    Offer practical sessions on assembling and cleaning pumps and related equipment.  Ensure milk expression equipment is readily available in designated milk expression rooms.  Ensure there are sufficient refrigerators for storing EBM in the NICU.  ***Instrumental support (Place to pump recommendations)***  Offer a range of pumping location options including during SSC/KMC, beside the incubator, or in a designated milk expression room.  Provide flexible SSC opportunities for both parents.  Ensure that parents of twins have opportunities for SSC contact with both infants.  Ensure parent input into the design and improvement of milk expression rooms.  ***Practical support: recommendations***  Ensure parents have access to scheduled expert lactation support for long term milk expression for a preterm baby across both hospital and community settings.  Appoint designated lactation consultants specifically dedicated to supporting parents in the NICU.  Ensure community LCs and PHNs meet with parents while the baby is in NICU and before discharge and that contact details are provided.  Ensure the continuation of specialist lactation support in community settings for parents of preterm VLBW infants. |
| **Theme 2: Navigating the Transition to Direct Feeding At Breast (DFAB)** | *Choosing whether to transition to DFAB* | “I was told from the start I was told they were premature there was no way I could breastfeed from the start that they would have to be that little bit older that they were being tube fed for the first while. And actually it was all of a sudden one day whatever particular nurse said do you want to try to breastfeed. I didn’t realise it was possible at that stage, but she said give it a try and there was no plan as such in place just give it a go. That was kind of it” (P7).  “The moment she came out, I wanted to breastfeed her straight away, that’s what I really wanted, but then they said she couldn’t be fed through the breast because she was too weak to do that” (P8).  “But they were very quick then to put the babies on the formula more, as soon as I had said ‘Oh, you know, when I was still in the phase of, will I, won't I… I don't know whether it's okay or not, and I wonder if they had encouraged me a bit more to try and keep with the breastfeeding, would I have done?” (P4).  “I was glad when she was finished with it; it took a lot of pressure off in terms of that commitment every day” (Partner 10). | Introduce the option of direct feeding at breast (DFAB) early in the neonatal intensive care journey as a sustainable and achievable feeding option for preterm VLBW infants.  Explore parents ’s knowledge and feelings about DFAB and provide personalised information that addresses their concerns.  Connect parents with other mothers who have successfully transitioned to DFAB.  Maintain an open dialogue about transitioning to DFAB and revisit this decision with parents.  Ensure that PHN or community-based lactation consultant discuss the mother’s feeding decisions and revisit the option of DFAB upon the baby’s discharge home. |
|  | ***Influencing factors in the transition to DFAB*** | ***Infant related factors***  “I think to be fair to people in the hospital, because obviously, they're so busy, they want to get the girls feeding and discharged, you know, which is perfectly normal, both sides want to get to keep the babies healthy and big enough to go home” (P3).  “But I had always been a bit sceptical about how much the kids would be getting, because obviously you don't know, your supply, you don't know. You know, you don't know the numbers. And that would drive me crazy” (P6)  ***Health system factors***  “When the girls were discharged a LC, did call to me a few times. She was helpful and showed me kind of, you know, best ways to breastfeed really, and to be fair, I did especially with the smaller twin, I did breastfeed her for the first few weeks nearly exclusively” (P3)  “I didn't get a visit from the lactation consultant into my home, but in fairness she did ring once or twice, definitely to see how I was” (P4).  “My public health nurse was actually a lactation consultant as well. So, they were telling me you know she would support you and to bring her out and she was, she was great. But he just couldn't take to it, we tried, you know, the tube as well and he was actually getting it from the bottle but on the breast kind of thing. But no, it just didn't suit him” (P5)  “The public health nurse, it was, she didn't have a lot of the answers for a premature baby. She didn't know. She didn't know what was normal with premature babies” (P6) | Co-design national and local DFAB guidelines with input from HCPs and parents to ensure relevance.  Develop and implement training programmes for hospital and community-based staff to support women with preterm infants in sustaining long-term milk expression and transitioning to DFAB.  Collaboratively develop a personalised DFAB transition plan with parents outlining realistic and achievable milestones for their baby’s progression in both hospital and community settings.  Ensure parents have access to scheduled expert lactation support in both hospital and community settings to promote DFAB.  Increase the number of NICU nurses and midwives with International Board Certified Lactation Consultant (IBCLC) qualifications.  Develop breastfeeding champion roles for nurses and midwives working in NICU’s.  Develop personalised feeding plans that address the emotional and logistical implications of one twin being discharged while the other twin remains in hospital.  Provide alternative feeding options to bottle feeding for VP infants in the NICU.    Offer opportunities for women to stay in the NICU prior to their baby’s discharge to support the transition to DFAB. |
| **Theme 3: The emotional Journey of feeding a preterm VLBW infant** | ***Subtheme 1: Processing an emotional infant feeding Journey*** | “At times when he got sick that I had to, when he was really unwell and you weren't expecting it. It's kind of hard you know, you are expected to come home and keep going at the same time. At the same time, it's probably the only thing that you could do for him. Yeah. Yeah. So that's, that keeps you going.” (P1)  “It was a massive focus (expressing breast milk). And it actually gave me a bit of sense of purpose in the first, especially the first while when it was very traumatic” (P2)  “I ended up quitting. And I do regret it. And I probably would, if I were to do my time again, I probably would have been a bit stronger about that. But sure Yeah. little regrets” (P6).  “The fact that I could (express), It probably helped us both, you know, in that regard. Just that something was coming from our side” (P3). | Inform parents about the range of emotional responses they may experience following a preterm birth, during long-term milk expression and when stopping expression and breastfeeding.  Share experiences from other parents who have engaged in long-term milk expression and transitioned to DFAB.  Regularly screen parents who have experienced the birth of a preterm VLBW infant for perinatal mental health symptoms in both NICU and community settings, using validated tools. |
|  | ***Sub theme 2***  ***Emotional support for infant feeding*** | “She (PHN) put me back in contact with the perinatal support, to be fair, I wouldn't like to say I wasn't open to going myself, it was never that I said that I don't need it. But I do think the public health nurse was great. And then the GP to be fair, she's actually very supportive as well, which is great” (P3).  “Well, I think what, everyone complimenting my supply was a big kind of a boost for me as, Oh yeah! that was great” (P5).  “But as well the pumping room where you know, you'd meet the other moms, I thought that was great as well that we could all kind of pump together and have a chat and tell our stories. That was a nice thing I really found” P5.  “There's nothing, there is absolutely nothing. And I've met a few people and they said that their partners found it really hard as well. That they could have done with having someone to talk to, someone in the same boat” (P6). | Train all healthcare professionals to provide empathetic and consistent emotional support to parents of VLBW infants, including encouragement and reassurance.  Provide information to parents about perinatal mental health supports in both hospital and community settings.  Facilitate peer connections between parents of preterm infants to help reduce feelings of isolation.  Implement training programs that support parents of VLBW infants adapt to their parental role, with specific guidance for parents of multiples. |

**Abbreviations:** Mothers own milk (MOM), Expressed Breast Milk (EBM), Very Low Birth Weight (VLBW), Very Preterm (VP), Lactation consultant (LC), Skin to Skin Contact (SSC), Public Health Nurse (PHN), Neonatal Intensive Care Unit (NICU), Direct Feeding At Breast (DFAB), Kangaroo Mother Care (KMC), Healthcare Professionals (HCPs)
